# Interactive Effects of Biosynthesized Nanocomposites and Their Antimicrobial and Cytotoxic Potentials

**DOI:** 10.3390/nano11040903

**Published:** 2021-04-01

**Authors:** Dina El-Kahky, Magdy Attia, Saadia M. Easa, Nemat M. Awad, Eman A. Helmy

**Affiliations:** 1 Microbiology Department, Faculty of Science, Ain Shams University, Cairo 11566, Egypt; sadiaeasa@hotmail.com; 2 Agricultural Microbiology Department, National Research Centre, 33 El-Bohouth Street, (Former El-Tahrir Street) Dokki, Giza 12622, Egypt; magdyattia3@gmail.com (M.A.); n_m_awad@yahoo.com (N.M.A.); 3 Microbiology Department, The Regional Center for Mycology and Biotechnology, Al-Azhar University, Cairo 11651, Egypt; emanhelmo@yahoo.com

**Keywords:** nanoparticles (NPs), metal nanocomposites (MNPCs), human pathogenic bacteria, antibacterial, antitumor, HCT-116 cells (colonic carcinoma)

## Abstract

The present study investigated the biosynthesis of silver (AgNPs), zinc oxide (ZnONPs) and titanium dioxide (TiO_2_NPs) nanoparticles using *Aspergillusoryzae*, *Aspergillusterreus* and *Fusariumoxysporum*. Nanocomposites (NCs) were successfully synthesized by mixing nanoparticles using a Sonic Vibra-Cell VC/VCX processor. A number of analytical techniques were used to characterize the synthesized biological metal nanoparticles. Several experiments tested biologically synthesized metal nanoparticles and nanocomposites against two types of human pathogenic bacteria, including Gram-positive *Staphylococcus aureus* and methicillin-resistant *Staphylococcus aureus* (MRSA), and Gram-negative *Escherichia coli* and *Pseudomonasaeruginosa*. Additionally, the antitumor activity in HCT-116 cells (colonic carcinoma) was also evaluated. Significant antimicrobial effects of various synthesized forms of nanoparticles and nanocomposites against *E. coli* and *P. aeruginosa* bacteria were detected. Various synthesized biogenic forms of nanoparticles and nanocomposite (9.0 to 29 mm in diameter) had high antibacterial activity and high antitumor activity against HCT-116 cells (colonic carcinoma) with IC_50_ values of 0.7–100 µg/mL. Biosynthesized NPs are considered an alternative to large-scale biosynthesized metallic nanoparticles and nanocomposites, are simple and cost effective, and provide stable nanomaterials.

## 1. Introduction

Biological synthesis of metal nanoparticles (NPs) is a new environmentally friendly approach in the context of green nanotechnology. The process of biological synthesis provides a wide range of environmentally acceptable methodologies and is cost effective, eco-friendly and very rapid [1]. These methods overcome the harmful effects on the environment caused by chemical synthesis. Biologically synthesized nanoparticles have many applications, such as catalysts of chemical reactions, biological labeling, antimicrobial agents, electrical batteries and optical receptors [2]. Microbial sources of nanoparticle production via metabolic activity are of great interest for nanoparticle precipitation.

Silver nanoparticles (AgNPs) have been widely used in the medical and cosmetic industries because of their unique antimicrobial properties [3]. AgNPs have a potent inhibitory effect against both Gram-negative (−ve) and Gram-positive (+ve) bacteria and yeasts on a large scale [4,5]. Recently, AgNPs were reported to destroy and damage the bacterial cell membrane; however, silver metal itself is considered a very good antibacterial agent [6,7]. Ag can destroy the structure of bacterial DNA; thus, respiratory enzymes in bacteria become inactivated due to the interaction of AgNPs with thiol groups; [8,9] additionally, AgNPs play an important role in the protection against human pathogenic bacteria, such as *Escherichia coli* (*E. coli*) and *Staphylococcus aureus* (*Staph. aureus*).

Zinc oxide is considered safe according to 21CFR182.8991 of the Food and Drug Administration of the United States of America (FDA) [10,11]. ZnO nanoparticles have enhanced antibacterial properties and efficiency, and effectively inhibit human pathogenic bacteria [12]. ZnONPs have great antibacterial effects against both Gram-positive (+ve) and Gram-negative (−ve) bacteria [13]. The antibacterial activity of ZnONPs increases concomitant to a decrease in particle size [14]. Development of zinc oxide-based therapeutic systems is expected to produce promising results [15].

Titanium dioxide is a metal that has special properties, such as good resistance to chemical erosion, nontoxicity, antimicrobial and anticancer effects [16]. TiO_2_ is attractive because it is stable, insoluble, nontoxic, resistant to corrosion and relatively inexpensive. Whether commercial or self-synthesized, TiO_2_ powders have significant variations in their structure, particle size and electronic properties that play a significant role in the photoactivity of TiO_2_. The photodegradation behaviors depend on the crystal structure of TiO_2_ [17].

Many strategies are available to overcome antibiotic resistance, including a reduction in the large use of antimicrobials, collection and analysis of the data, avoidance of inappropriate use of antimicrobial agents in farm animals, and development of new drugs and nanotechnology [18]. The development of nanotechnology has resulted in the synthesis of nano-sized organic and inorganic molecules with potential applications in textiles, medicine, food packaging, industry, and therapeutics. The development of new nano-sized antibacterial agents and nanocomposites can be an alternative strategy used to overcome antimicrobial resistance [18]. AgNPs have limited applications in the human body and have toxic effects on human cell lines [6]. Recently, silver has been shown to have a significant enhancing effect on the antibacterial activity of titanium dioxide because of the synergistic effect of nano-silver [7].

Ag-NPs and ZnO-NPs have been proved to have effective antimicrobial activity at very low concentrations even to resistant strains of microbes [19]. This is because they destroy many biological pathways in a cell and it would necessitate the microorganism to go through many concurrent transmutations to develop resistance [20]. Furthermore, they need very short contact time to cause a long-lasting cell growth inhibition [21]. Recently, the Ag/ZnO nanocomposite has been found to show enhanced antimicrobial activity against common microbes compared to the individual nanoparticles [22]. This is because the properties of the hybrid are not just the sum of the individual advantages of both nanoparticles but are derived from their synergic effect which creates a new class of hybrid-nanomaterials [23].

Bactericidal properties of silver-titanium dioxide nanocomposites in killing bacteria and viruses have been reported [24]. Metallic silver increases the photocatalytic activity of conjugated titanium dioxides, thereby decreasing the band gap of titanium dioxide to decrease the recombination of the charge carriers photoexcited from the valence point to the conduction band of titanium dioxide.

The degradation of organic pollutants is due to photoexcited charge carriers, and degradation of organic pollutants is specifically caused by holes, which act by forming high levels of hydroxyl radicals [25]. The presence of silver within titanium dioxide increases the antibacterial activity because of cell wall and membrane damage by AgNPs [26]. The Ag-TiO_2_ nanocomposites are advantageous for several reasons: (a) the antimicrobial effect and potential toxicity of titanium dioxide to normal cells are low because of the presence of a very low nontoxic concentration of silver, and titanium dioxide shows no cytotoxicity in the human body or cells; (b) strong photocatalytic effect-dependent removal of organic pollutants from wastewater; and (c) ability to destroy bacteria found in contaminated wastewater. The photocatalytic and antibacterial effects of TiO_2_-Ag nanocomposites have been documented in some studies; however, characterization of their structure and microstructure was not described [27,28]. The mechanism of action was not clearly discussed in recent studies [27]. To enhance the photocatalytic performance of various morphological shapes of nanocomposites, such as nanotubes, nanosheets were prepared as documented in some previous studies [29]. The synergistic activity of silver in the titanium dioxide matrix produces a potentially antibacterial material. In almost all cases, nanocomposite synthesis is considered to be highly complex because it requires expensive chemicals and several steps are time-consuming [30,31].

In this study, nanocomposites were synthesized by fast and facile mechanical alloying. Various biosynthesized nanoparticles and nanocomposites with antibacterial properties and antitumor efficiency were produced and can be applied in several biomedical products, which use metals nanoparticles (MNPs) and metal nanocomposites (MNPCs) for electrode modification.

## 2. Materials and Methods

Three isolates of *Aspergillusoryzae*, *Aspergillusterreus* and *Fusariumoxysporum* were isolated from soil and selected due to their growth and production of nanoparticles [32]. The isolates were selected based on the high efficiency for biosynthesis nanoparticles.

### 2.1. Biosynthesis of Silver (AgNPs), Zinc Oxide (ZnONPs) and Titanium Dioxide (TiO_2_NPs) Nanoparticles

For biosynthesis, the biomass of fungal cells was prepared, and fungi were aerobically grown in modified liquid Czapeck Dox medium (sucrose 30 g, NaNO_3_ 2.0 g, KH_2_PO_4_ 1 g, MgSO_4_ × 7H_2_O 0.5 g, KCl0.5 g and FeCl_3_ × 6H_2_O 0.001 g [33]) in 250 mL Erlenmeyer flasks containing 100 mL medium for the preparation of AgNPs and ZnONPs; TiO_2_NPs were prepared in malt extract broth (malt extract 30 g and peptone 5 g, pH 5.4 ± 0.2 [34]). The inoculated fungal isolates in the flasks were agitated at 150 rpm and incubated on an orbital shaker at 28 °C. Fungal biomass was harvested using a plastic sieve, and Milli-Q deionized water was used for extensive washing to remove any extra medium components from the fungal biomass. Fresh fungal biomass (20 g) was mixed with 100 mL of Milli-Q deionized water for 72 h at 28 °C in an Erlenmeyer flask and washed by agitation. The cell filtrate was collected after incubation by passing through Whatman filter paper No 1. The production of nanoparticles AgNPs, ZnONPs and TiO_2_NPs was performed as reported previously [14,35,36,37]. For synthesis of AgNPs, ZnONPs and TiO_2_NPs, zinc oxide (10 mM ZnO), titanium dioxide (5 mM TiO_2_) and silver nitrate (1.5 mM) were mixed with 50 mL of the fungal cell filtrate in a 250 mL Erlenmeyer flask and agitated in an orbital shaker for 5 min. A flask containing the control fungal cell filtrate without the metal ions was used as a control.

### 2.2. Preparation of Nanocomposites

Nanoparticles were mixed as shown in Table 1; 700 µg of AgNPs was added to 10 mL of double deionized water (DDW); 1400 µg of ZnONPs was added to 10 mL DDW, and 800 µg of TiO_2_NPs was added to 10 mL DDW. Then, the particles were mixed using a Sonic Vibra-Cell VC/VCX 750 processor at (NRC) National Research Centre, Cairo, Egypt; the samples were examined using UV spectrophotometry, FTIR, and TEM before and after sonication.

### 2.3. Characterization of Biosynthesized AgNPs, ZnONPs, TiO_2_NPs and Nanocomposites

The properties of AgNPs, ZnONPs, TiO_2_NPs and nanocomposites were determined based on ultraviolet–visible absorption spectra (UV-2401 PC) at wavelengths ranging from 200 to 800 nm. For FTIR spectroscopy analysis, vacuum-dried AgNPs, ZnONPs, TiO_2_NPs and nanocomposites were mixed with potassium bromide (KBr) at a ratio of 1:100, and the spectra were recorded by a Shimadzu 8400S instrument. Scanning data were acquired from the average of 49 data scans within the range from 4000 to 400 cm^−1^ to identify the functional groups involved in metal reduction and in the synthesis of nanoparticles and nanocomposites. The liquid reaction mixture after bioreduction was dried at 45 °C in a vacuum drying oven. Then, the dried mixture was collected for the determination of the formation of nanoparticles and nanocomposite. The purity phase, structure and crystallinity of the dried nanoparticles and nanocomposites were determined by analyzing their XRD patterns (Philips Analytical X-ray) at 30 kV, 20 mA with Cu Kα radiation. The spectra were analyzed, recorded and processed by WIN-FIT software [38]. The nanoparticles and nanocomposites were used to prepare a film on a carbon-coated copper grid for transmission electron microscopy and were analyzed at a voltage of 120 kV by a transmission electron microscope (Tecnai 120) [39].

### 2.4. Evaluation of Antibacterial Activity

Bacteriostatic activities were determined using four strains; the isolates being selected considering their availability, for measuring the antimicrobial effect including Gram-negative (−ve) bacteria *Escherichia coli* O157H7 and *Pseudomonas aeruginosa* ATCC10145 and Gram-positive (+ve) bacteria *Staphylococcus aureus* NRC 23,516 and methicillin-resistant *Staphylococcus aureus* (MRSA) NRC 629012. The isolated cultures were provided by the Agricultural Microbiology Department, National Research Centre, Cairo, Egypt. All bacterial strains were stored as frozen stocks at −80 °C (Protect Bacterial Preservation System, Technical Service Consultants) and maintained on Luria agar (LA) medium at 37 °C. Ampicillin antibiotic sensitivity disks were used to test the effect of a particular antibiotic on various bacterial cultures; each disk contained 10 µg ampicillin, which decreased the chance of developing resistance to antibiotics by bacterial strains. The bacteriostatic activities of nanoparticles were investigated by the disc diffusion method [40]. LB agar media were prepared in plates, sterilized and solidified; then, the bacterial cultures were plated using a swab. MNPs and nanocomposites (NCs) solutions at various concentrations (6.25, 12.5, 25, 50 and 100 μL) were placed in agar plates, which were incubated at 37 °C for 24 h. The inhibition zones were identified, measured and compared with the control samples (cell-free water extract).

### 2.5. Determination of the Antitumor Activity

HCT-116 (colonic carcinoma) cells were obtained from the VACSERA Tissue Culture Unit, Dokki, Egypt. Crystal violet staining was used to determine the cytotoxicity of the synthesized NPs and NCs in HCT116 tumor cells. Approximately 1 × 104 cells were added to each well of 96-well culture plates for 24 h and incubated in a humidified atmosphere of 95% air and 5% CO_2_. The biosynthesized NPs and NCs were added at 0.2–100 μg/mL concentrations to HCT116 cells and incubated for 36 h. The control cells were treated with DMSO. Twenty microliters of crystal violet solution were added to each well for staining after the incubation, and the samples were incubated for an additional 4 h. Then, 200 μL DMSO was added. For the crystal violet assay, cells were stained at room temperature by 0.5% crystal violet (Sigma-Aldrich Corp., St. Louis, MO, USA) in 30% ethanol for 10 min. Cells were lysed by adding 1% SDS (sodium dodecyl sulfate) solution. The absorbance of the solution was determined using an ELISA plate reader at a wavelength of 595 nm [40]. To assess the potency of the antitumor activity of the compounds, the IC_50_ values were calculated as a concentration that inhibited cell viability corresponding to 50% viability of the control cells. The IC_50_ values (the half maximal inhibitory concentration) were calculated based on the best fit (R_2_ > 0.95) R_2_ = the square of the correlation of the Hill slope curve by nonlinear regression analysis of experimental data using Graph Pad Prism (version 5, Graph Pad Software, Inc., La Jolla, USA) according to the equation Y = 100/(1 + 10^((X − LogIC_50_) where (X) is log of dose, (Y) is the value of growth inhibition normalized to the control, and Hill Slope is a unitless slope factor [41].

Cell Viability was determined as follows [41]:

Cell Viability (%) = (Abs S/Abs C) × 100

where Abs S is the absorbance of the cells treated with NPs or NC and Abs C is the absorbance of untreated cells (control).

### 2.6. The statistical Analysis

All statistical analyses were performed using the SPSS (Statistical Package for the Social Sciences) software package (SPSS Inc., Routledge, NY, USA) and Excel (Microsoft, Routledge, NY, USA). The resulting data were used for the analysis of variance, and significant differences between the mean values were evaluated by using Duncan’s multiple range tests (*p* < 0.05).

## 3. Results

### 3.1. The UV–Vis Spectra

**Figure 1:**
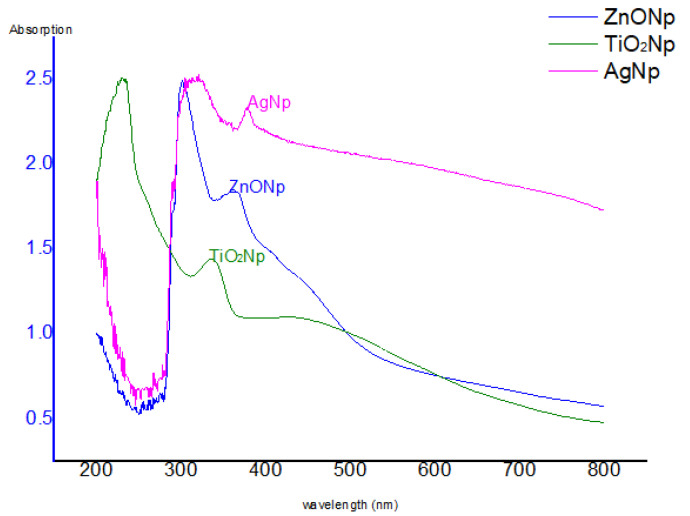
Ultra-violet visible spectrophotometer recorded after 72 h of reactions of solution of AgNPs, ZnONPsand TiO_2_NPs.

**Figure 2:**
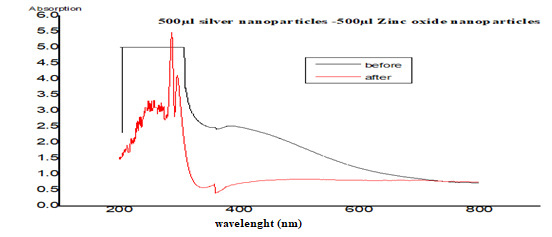

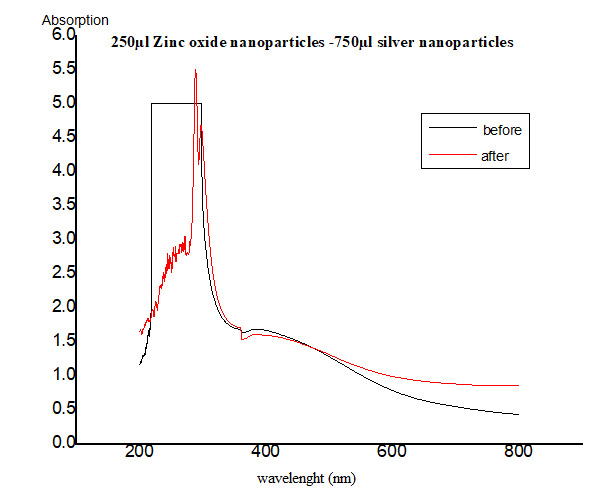
Ultra-violet visible spectra recorded of nanocomposites before and after sonication.

The ultraviolet absorption spectra were recorded after 72 h of the reaction of the cell filtrate with silver nitrate, zinc oxide and titanium dioxide metal ions (Figure 1). Extinction peaks were identified at approximately 420 nm for AgNPs, 377 nm for ZnONPs, and 360 nm for TiO_2_NPs. The reaction was evaluated after 72 h of incubation, and the peak intensity demonstrated an increase in the reaction progress corresponding to an increase in the number of NPs. The spectra of 14 nanocomposites were recorded before and after sonication; the results of Figure 2 show that the best NCs production was achieved by the composites of 500 μL AgNPs with 500 μL ZnONPs, 750 μL AgNPs with 250 μL ZnONPs, and 750 μL TiO_2_NPs with 250 μL ZnONPs, which had clear peaks on the UV spectra; the remaining nanocomposites did not show any peaks (not shown). These three nanocomposites were selected for further study based on the results of UV spectroscopy.

### 3.2. FTIR Spectrum of Nanoparticles

**Figure 3:**
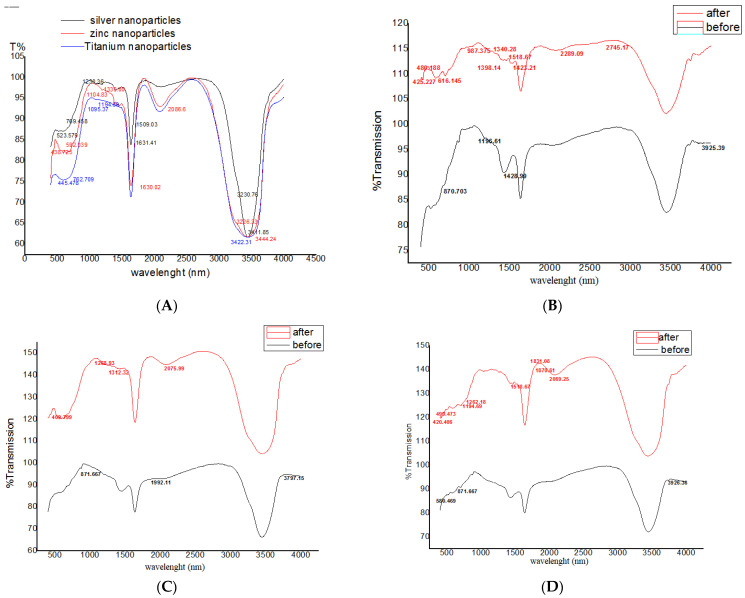
FTIR of (**A**) AgNPs, ZnONPs and TiO_2_NPs; (**B**) composite 500 μL AgNPs–500 μL ZnONPs; (**C**) composite 750 μL AgNPs–250 μL ZnONPs; (**D**) composite 750 μL TiO_2_NPs–250 μL ZnONPs before and after.

Figure 3A shows FTIR spectral analysis of AgNPs, ZnONPs and TiO_2_NPs. The FTIR spectrum of AgNPs showed a band at 3411 cm^−1^ assigned based on the characteristics of the hydroxyl groups and surface-adsorbed water. Appearance of the absorbance peaks at 3230 cm^−1^ corresponded to the stretching vibration of the NH stretching of Amide A. Additionally, the bands at 1631 and 1509 cm^−1^ corresponded to the stretching vibrations of C=C and Amide II. The absorption bands at 523 cm^−1^ corresponded to the stretching vibration of the oxygen-stretched bond.

FTIR for ZnONPs the groups at 3444 cm^−1^ were assigned to the surface-adsorbed water and the hydroxyl moiety of the ZnONPs groups. An absorbance peak at 3226 cm^−1^ corresponded to the stretching vibration of the N-H stretching bond, and a peak at 1458 cm^−1^ corresponded to the bending vibrations of –COO. A peak at 1385 cm^−1^ was assigned to the bending vibrations of the residual NO_3_^−^. The absorption peaks at 592 and 438 cm^−1^ corresponded to the bending vibrations of =C-H bending and oxygen-stretched bonds.

A band in the FTIR spectrum of TiO_2_NPs at 3422 cm^−1^ was assigned to the stretching vibration of the hydroxyl group (O-H stretching) of TiO_2_NPs, and the bands at 1638 cm^−1^ corresponded to the bending vibrations of the N-H bond because of dihydrogen titanate phase formation. A peak at 1384 cm^−1^ indicated the formation of amide linkages, and a peak at 1095 cm^−1^ corresponded to the stretching vibration of C=C. The absorption bands at 762 and 445 cm^−1^ corresponded to the =C-H bending and oxygen-stretched bonds, respectively.

The FTIR spectrum analysis of composite nanoparticles of 500 μL AgNPs with 500 μL ZnONPs prepared without sonication with Sonic Vibra-Cell showed intense absorption bands at 870 cm^−1^ assigned to the C-N-C ring bending; a peak at 1196 cm^−1^ corresponded to the C-O stretching bond; the bands at 1428 and 3925 cm^−1^ were assigned to the C-C ring vibration stretching and N-H stretching (Amide A band), respectively. Treatment with Sonic Vibra-Cell resulted in appearance of intense absorption bands at 425 and 480 cm^−1^ corresponding to the -C stretching (Figure 3B).

A peak at 616 cm^−1^ was assigned to the C-C=O bond bending; a peak at 987 cm^−1^ was assigned to the O stretching vibration; the peaks at 1340 cm and 1398 cm^−1^ were assigned to aliphatic C-H bending and scissoring of CH_3_ and CH_2_ and C-H bending, respectively, and a peak at 2745 cm^−1^ was assigned to the aldehyde C-H bending. FTIR spectrum analysis of the composite nanoparticles formed by 750 μL AgNPs with 250 μL ZnONPs without treatment with Sonic Vibra-Cell showed intense absorption bands at 871, 1992 and 3767 cm^−1^ corresponding to the C-N-C ring bending, -N=C=N-, -N3, C=C=O, and NH and OH stretching, respectively. Treatment with Sonic Vibra-Cell resulted in appearance of intense absorption bands at 409, 1268, 1312 and 2075 cm^−1^ corresponding to the -C stretching, Amide III, C-N stretching of aromatic amine and C≡C stretching that was not present in symmetrical alkynes (Figure 3C).

FTIR spectrum analysis of composite nanoparticles formed by 750 μL TiO_2_NPs with 250 μL ZnONPs without treatment with Sonic Vibra-Cell showed intense absorption bands at 580, 871 and 3926 cm^−1^ corresponding to the hydroxyl group of the O-H compounds, C-N-C ring bending and N–H stretching bands (Amide A band), respectively. Treatment with Sonic Vibra-Cell resulted in appearance of intense absorption bands at 420 and 499 cm^−1^ associated with the -C stretching. A peak at 1194 cm^−1^ was assigned to C-O stretching; a peak at 1262 cm^−1^ corresponded to Amide III; the peaks at 1518.67, 1870.61 and 2069.25 cm^−1^ in Figure 3D corresponded to the absorption bands associated with CH2 and CH3, C=O and N=C=S, respectively.

### 3.3. Analysis of Transmission Electron Microscope Images

**Figure 4:**
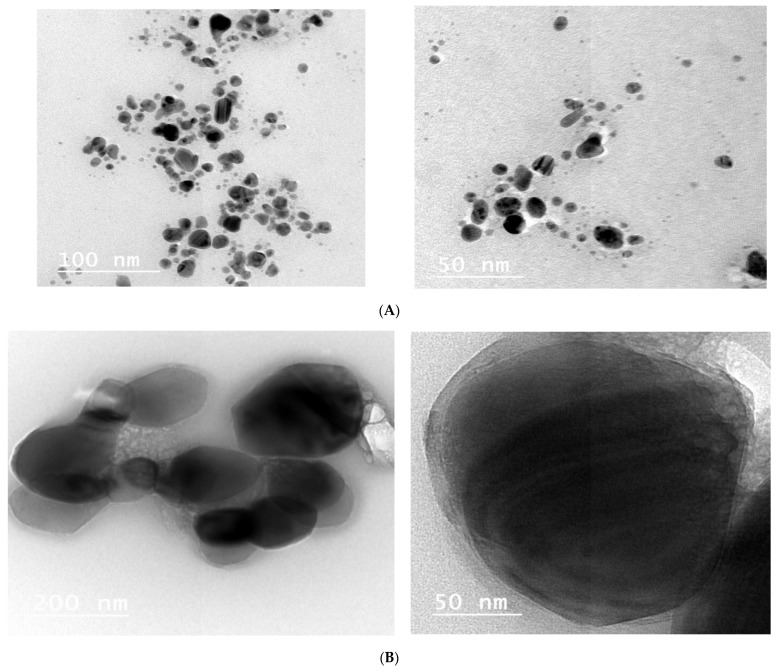

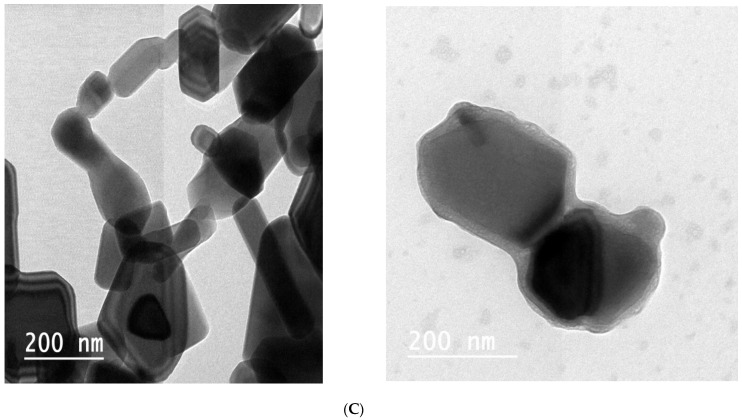
TEM micrograph (**A**) AgNPs, (**B**) ZnONPs, (**C**) TiO_2_NPs.

The transmission electron microscopy images (Figure 4A–C) indicated that nanoparticles are spherical in shape with a wide range of size distributions. The larger particles appeared to consist of a number of small particles in an agglomerated shape. TEM microscopy images of AgNPs are shown in Figure 4A. The size of nanoparticles was in the range of approximately 5–30 nm; the spherical regular shape had smooth surfaces; and the distribution frequency included nanoparticles in the 10–15 nm range. TEM images of ZnONPs are shown in Figure 4B. The nanoparticles had regular cubic or spherical shape with smooth surfaces within approximately 70–130 nm size; almost all nanoparticles were 110 nm in size. On the other hand, the TEM image in Figure 4C shows that the TiO_2_NPs had a regular spherical shape with smooth surfaces and a size distribution within the range of approximately 50–90 nm. TEM shows that almost all particle sizes were 70 nm.

**Figure 5:**
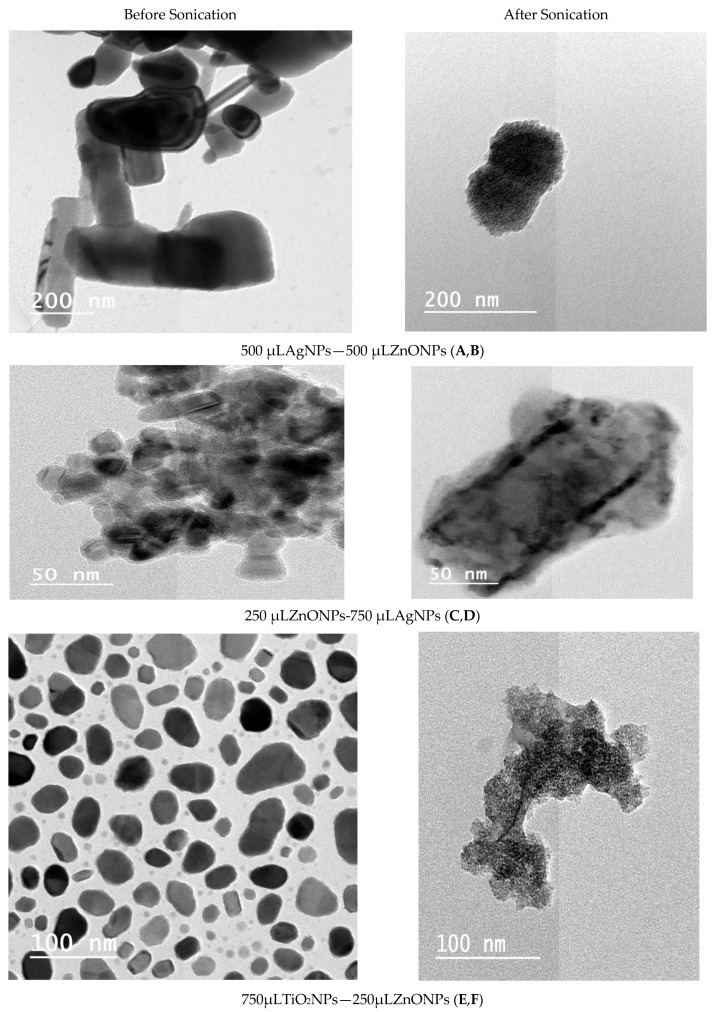
TEM micrograph (**A**,**B**) composite 500 μL AgNPs–500 μL ZnONPs; (**C**,**D**) composite 750 μL AgNPs–250 μL ZnO nanoparticles; (**E**,**F**) composite 750 μL TiO_2_ nanoparticles–250 μL ZnO nanoparticles.

Figure 5 shows a diversity of the particle sizes of various composite nanoparticles before and after sonication. Additionally, the TEM images suggest that the particles were poly compact after sonication. The images of 500 μL AgNP–500 μL ZnONPs in Figure 5A,B show a polyhedral shape of Ag with ZnO dots on the surface of the particles. After sonication of TiO_2_ and ZnO NCs, the nanorods and flakes in zinc oxide were changed to a spherical shape, as shown in Figure 5E,F. A typical transmission electron microscopy image of the titanium dioxide–zinc oxide nanocomposite material was very similar to that of titanium dioxide and zinc oxide and looked like a sandwich. Similarly, transmission electron microscopy showed high-magnification images of the titanium dioxide-zinc oxide nanocomposite.

### 3.4. X-Ray Diffraction (XRD) Pattern Analysis

**Figure 6:**
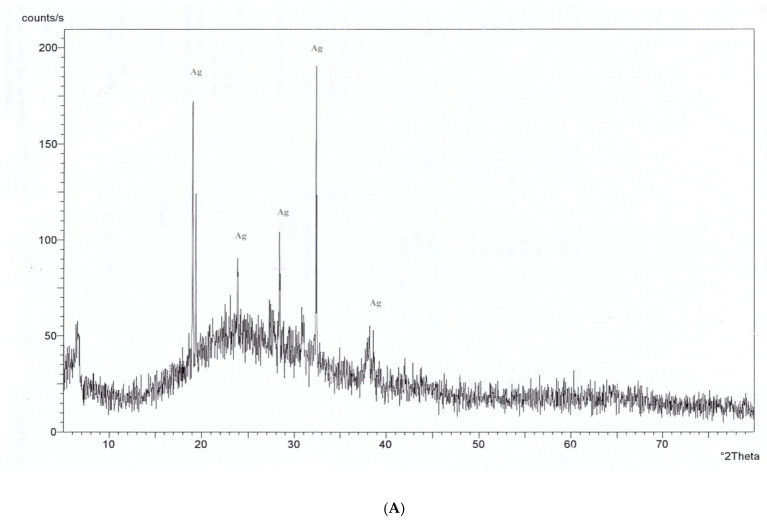

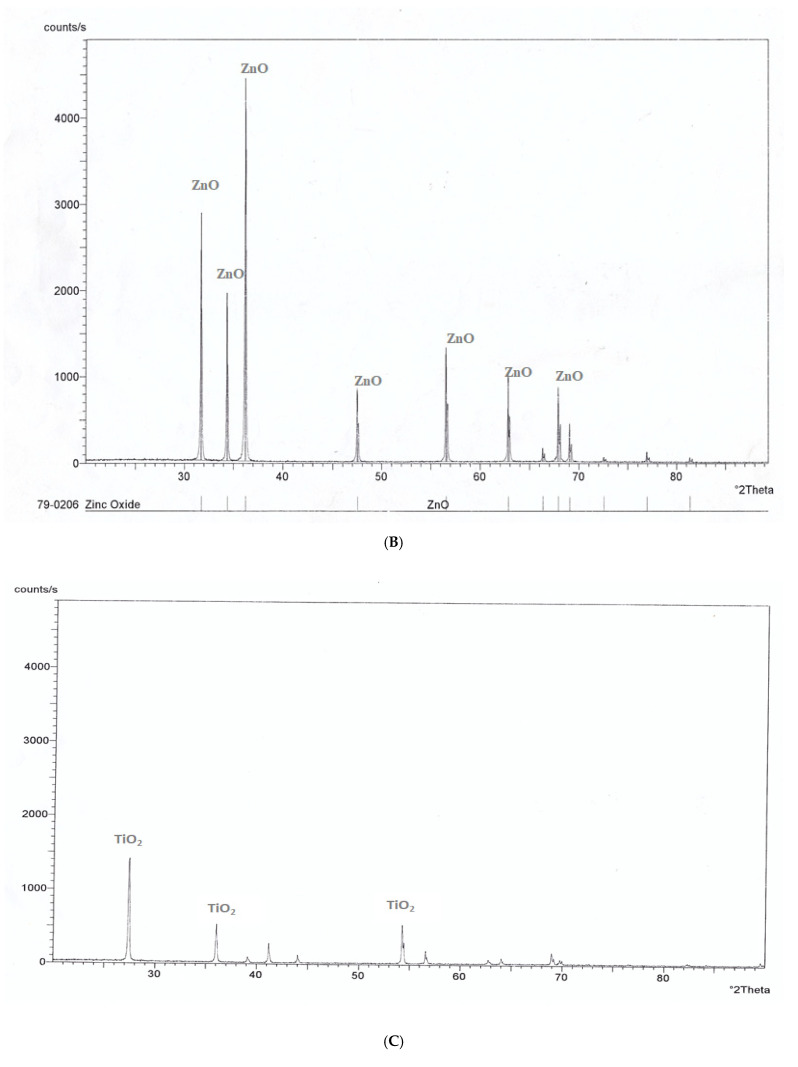
X-ray diffraction pattern of AgNPs (**A**), ZnONPs (**B**), TiO_2_NPs (**C**) and diffraction pattern of composite nanoparticles (**D**) composite 500 μL AgNPs–500 μL ZnO nanoparticles, composite 750 μL AgNPs–250 μL ZnO nanoparticles and composite 750 μL TiO_2_ nanoparticles–250 μL ZnO nanoparticles.

Phase purity and composition of the particles were examined by XRD; Figure 6A shows typical XRD pattern of AgNPs appeared in the range of 20–80° at a scanning step of 0.01. A number of Bragg reflections with 2θ values of 19.017°, 28.348°, 30.894°, 32.337°, 38.079° were observed corresponding to (001), (001), (003), (001) and (002) planes, documenting a typical XRD pattern of AgNPs in the range of 0–80° with Philips Analytical X-Ray. The phase purity and composition of the ZnONPs examined by XRD shows a typical XRD pattern of ZnONPs appeared in the range of 30–80° at a scanning step of 0.01 [42]. Figure 6C shows a typical XRD pattern of TiO_2_NPs appeared in the range of 30–80°. A number of Bragg reflections with 2θ values of 27.389°, 36.043°, 41.205°, 44.007°, 54.425°, 56.734°, 68.960° and 69.965° were observed corresponding to (101), (002), (002), (001), (001), (001), (001) and (002) planes, showing a typical XRD pattern of TiO_2_NPs in the range of 30–80° with Philips Analytical X-Ray. Figure 6D shows a typical XRD pattern of different nanocomposing (500 μL AgNPs–500 μL ZnONPs, 250 μL ZnONPs–750 μL AgNPs and 250 μL ZnONPs–750 μL TiO_2_NPs). Regarding nanocomposing 500 μL AgNPs–500 μL ZnONPs, Figure 6 shows that a number of Bragg reflections with 2θ values of 31.751°, 34.406° and 36.235° were observed corresponding to (001), (001) and (001) planes. However, in the nanocomposing 250 μL ZnONPs–750 μL AgNPs, a number of Bragg reflections with 2θ values of 31.7874°, 34.442° and 36.271° were observed corresponding to (001), (001), and (002) planes, and, in the case of nanocomposing 250 μL ZnONPs–750 μL TiO_2_NPs, in the range of 0–80°. A number of Bragg reflections with 2θ values of 31.810°, 34.463° and 36.289° were observed corresponding to (001), (002), and (002) planes with Philips Analytical X-Ray.

### 3.5. Application Studies of the Produced Nanoparticles

#### 3.5.1. Comparative Toxicity Study of the Biosynthesized Nanoparticles and Nanocomposite on the Human Bacterial Diseases

Biosynthesized NPs and capped NC produced inhibition zones in Gram-negative bacteria (*Escherichia coli* O157H7 (*E. coli*) and *Pseudomonas aeruginosa* ATCC10145 (*P. aeruginosa*)), and Gram-positive bacteria (*Staphylococcus aureus* NRC23516 (*Staph. aureus*) and methicillin-resistant *Staphylococcus aureus* NRC629012 (MRSA)). The antimicrobial activity was assayed on LBA plates using the paper-disc method.

#### 3.5.2. Antimicrobial Effect of Nanoparticles and Nanocomposite

**Figure 7:**
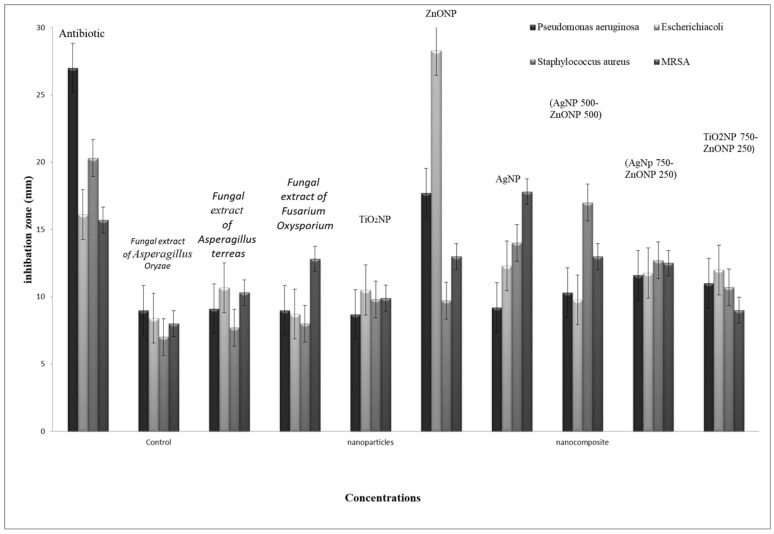
The inhibition zones of biosynthesized nanoparticles and capping nanocomposite. SD±, standard deviation.

The bacteriostatic activity assay of ZnONPs, TiO_2_NPs, AgNPs and NCs is shown in Figure 7. Various concentrations (6.25, 12.5, 25, 50 and 100 μL) were placed on the discs, and inhibition zones of various pathogenic microbes were measured. Figure 7 shows that, in general, 750 μL TiO_2_NPs–250 μL ZnONPs had the lowest activity towards bacteria with a limited inhibition zone. Antibacterial activity results revealed that ZnONPs, AgNPs and their composites are excellent antibacterial agents against both Gram-positive and Gram-negative bacteria. The result in Figure 7 also shows that NCs were better than nanoparticles in inhibiting the growth of *P. aeruginosa* at all concentrations used (100, 50, 25, 12.5 and 6.25 μL), with values 39.4%, 46.5%, 22.2%, 11%, and 11% greater, respectively, in the case of nanocomposite (750 μL AgNP–250 μL ZnONPs) compared to ZnONPs nanoparticles. Results also show that NCs were better than nanoparticles in inhibiting the growth of *Escherichia coli* at all concentrations used (100, 25, 12.5 and 6.25 μL), with values 48.9%, 28.2%, 17.11% and 33.3% greater, respectively, in the case of nanocomposite (750 μL AgNP–250 μL ZnONPs) compared to ZnONPs nanoparticles; at 50 μL, nanoparticles were 13.3% better than the nanocomposite. The inhibiting zones of *Staph.aureus* at all concentrations used (100, 25, 12.5 and 6.25 μL) were 30%, 28.2%, 17.11%, and 33.3% greater, respectively, in the case of nanocomposite (750 μL AgNP–250 μL ZnONPs) compared to ZnONP nanoparticles; at 50 μL nanoparticles were 13% better than the nanocomposite. The growth of MRSA at all concentrations used (50, 25, 12.5 and 6.25 μL) were 4%, 5.7%, 30%, and 133% greater, respectively, in the case of the nanocomposite (750 μL AgNP–250 μL ZnONPs) compared to ZnONPs nanoparticles; at 100 μL nanoparticles were 17.9% better than the nanocomposite.

**Figure 8:**
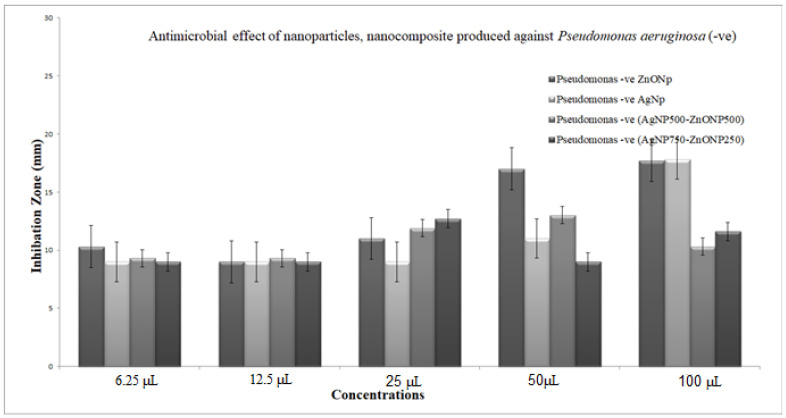
The antimicrobial activity of the nanoparticles and nanocomposite produced against *Pseudomonas aeruginosa* showing increasing inhibition zone with increasing amounts of nanoparticles: 6.25, 12.5, 25, 50 and 100 μL with volume made up to 100 μL with distilled water wherever needed. Zinc oxide nanoparticles (ZnONPs); silver nanoparticles (AgNPs); composite 500 μL AgNPs–500 μL ZnONPs; composite 750 μL AgNPs–250 μL ZnONPs nanoparticles. SD±, standard deviation.

**Figure 9:**
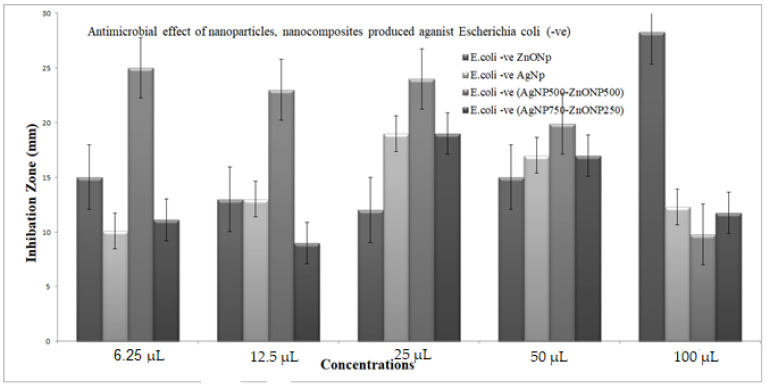
The antimicrobial activity of the nanoparticles and nanocomposite produced against *Escherichia coli*. Data showing increasing inhibition zone with increasing amounts of nanoparticles: 6.25, 12.5, 25, 50 and 100 μL with volume made up to 100 μL with distilled water wherever needed. Zinc oxide nanoparticles (ZnONPs); silver nanoparticles (AgNPs); composite 500 μL AgNPs–500 μL ZnONPs; composite 750 μL AgNPs–250 μL ZnONPs nanoparticles. SD±, standard deviation.

**Figure 10:**
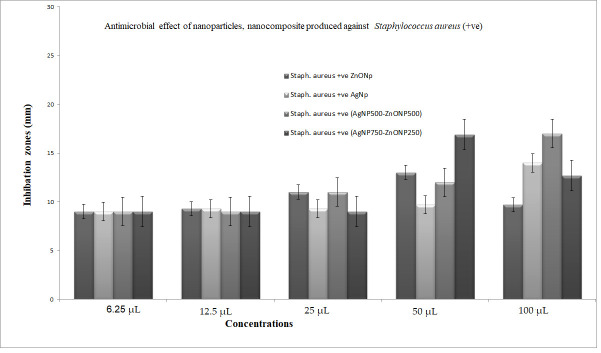
The antimicrobial activity of the nanoparticles and nanocomposite produced against human pathogenic microbe: *Staphylococcus aureus*. Data showing increasing inhibition zone with increasing amounts of nanoparticles: 6.25, 12.5, 25, 50 and 100 μL with volume made up to 100 μL with distilled water wherever needed. Zinc oxide nanoparticles (ZnONPs); silver nanoparticles (AgNPs); composite 500 μL AgNPs–500 μL ZnONPs; composite 750 μL AgNPs–250 μL ZnONPs nanoparticles. SD±, standard deviation.

**Figure 11:**
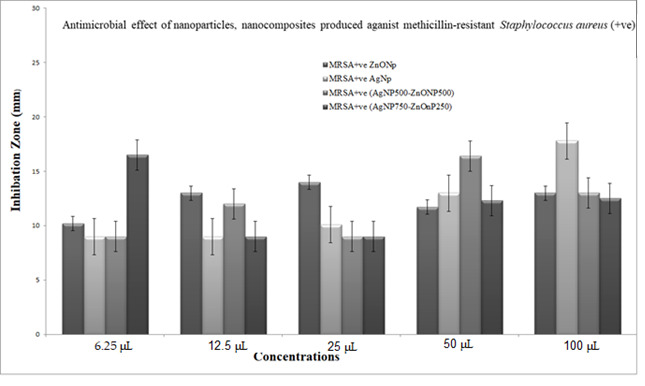
The antimicrobial activity of the nanoparticles and nanocomposite produced against human pathogenic microbe: MRSA sp. Data showing increasing inhibition zone with increasing amounts of nanoparticles: 6.25, 12.5, 25, 50 and 100 μL with volume made up to 100 μL with distilled water wherever needed. Zinc oxide nanoparticles (ZnONPs); silver nanoparticles (AgNPs); composite 500 μL AgNPs–500 μL ZnONPs; composite 750 μL AgNPs–250 μL ZnONPs nanoparticles. SD±, standard deviation.

Figure 8, Figure 9, Figure 10 and Figure 11 shows that most of the tested microbes were inhibited at low concentrations of the nanoparticles (6.25 μL), and the inhibition zones gradually increased with increasing concentrations of ZnONPs, AgNPs, composite 500 μL AgNPs–500 μL ZnONPs and composite 750 μL AgNPs–250 μL ZnONPs in the case of *E. coli*, *Pseudomonas aeruginosa*, *Staphylococcus aureus*, and MRSA (Figure 8). It was also observed that at concentrations of 100 and 50 μL, the zones became wider than those at other concentrations. In addition, the inhibition zones caused by nanoparticles and NCs were gradually increased in the case of *E. coli* and *P. aeruginosa* (Gram-negative bacteria), as shown in Figure 8 and Figure 9 compared with those detected in the case of *Staph. aureus* and MRSA (Gram-positive bacteria), which are illustrated in Figure 10 and Figure 11.

#### 3.5.3. Antitumor Activity of Nanoparticles and Nanocomposites

Cytotoxic activity of nanoparticles was further tested in vitro by determining the number of surviving cells by staining HCT-116 cells (colonic carcinoma) with crystal violet; cell viability was determined after cultivation with nanoparticles for 24 h, and a control (no nanoparticles) was used for comparison.

**Figure 12:**
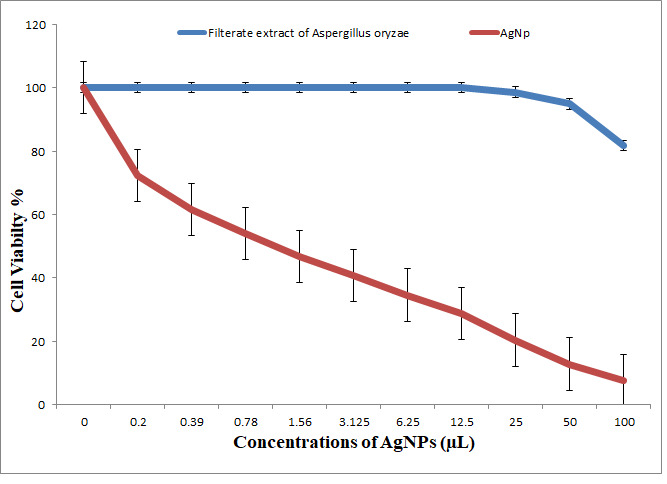
Viability of HCT-116 cells (colon carcinoma cell) (24 h incubation). Treatment with cell filtrate of *Aspergillusoryzae* as control and treatment with silver biosynthesized nanoparticles (AgNPs).

**Figure 13:**
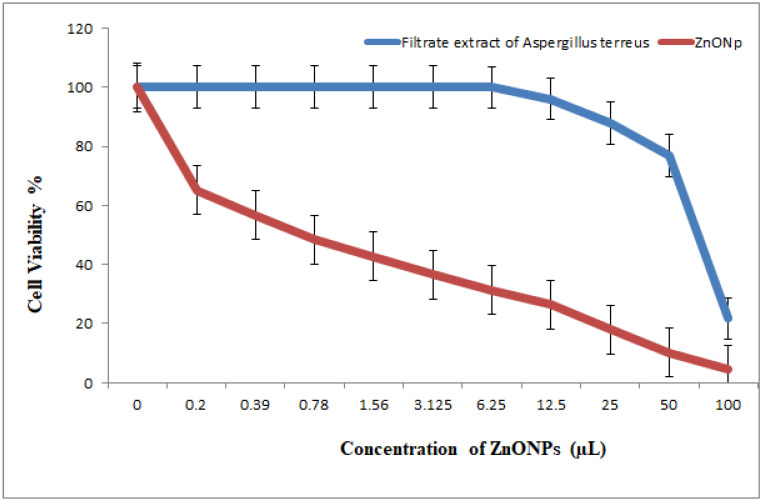
Viability of HCT-116 cells (colon carcinoma cell) (24 h incubation). Treatment with cell filtrate of *Asperagillusterreus* as control and treatment with zinc oxide biosynthesized nanoparticles (ZnONPs).

**Figure 14:**
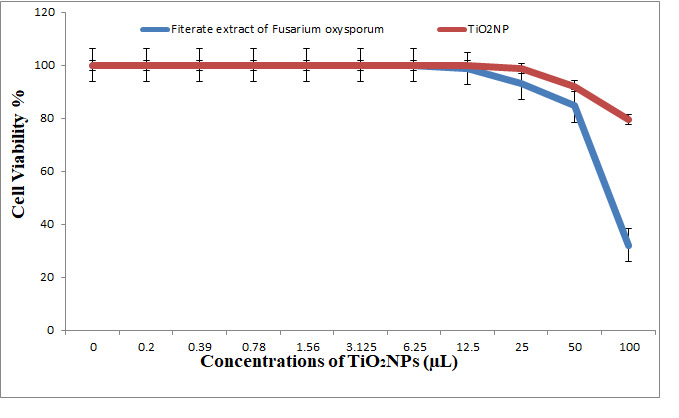
Viability of HCT-116 cells (colon carcinoma cell) (24 h incubation). Treatment with cell filtrate of *Fusariumoxysporium* as control and treatment with titanium dioxide biosynthesized nanoparticles (TiO_2_NPs).

The results indicated antitumor activity of biosynthesized NPs and NCs against the HCT-116 colonic carcinoma cell line; the cytotoxicity was increased concomitant to an increase in the concentrations of metal nanoparticles. Cell viability was dose-dependently decreased at a very low concentration (IC_50_ = 1.2 μL) in the case of AgNPs; the half maximal inhibitory concentration was characterized by IC_50_ => 100 μL in the case of the *Aspergillusoryzae* control (no nanoparticles) (Figure 12, Figure 13 and Figure 14). The results indicated that AgNPs selectively inhibited cancer cells. Cell viability was dose-dependently decreased at a very low concentration (IC_50_ = 0.7 μL) of ZnONPs; the control *Aspergillusterreus* (without nanoparticles) had an IC_50_ = 74.4 μL. At 25 μL, the inhibition became 0%, and the viability of HCT-116 tumor cells became 100% (Figure 12, Figure 13 and Figure 14). Cytotoxic effects of synthesized TiO_2_NPs against cancer cell lines have been limited [40].

In our study, crystal violet staining was used to evaluate the effect of TiO_2_NPs on HCT-116 cells (colonic carcinoma), and *Fusariumoxysporum* was used as a control. Dose-dependent cytotoxicity was detected in TiO_2_NP-treated HCT-116 cancer cells. The IC_50_ of TiO_2_NPs against HCT-116 cells was >100 μL, whereas the IC_50_ in the control samples was 82.9 μL (Figure 14). Our results also indicated that synthesized TiO_2_NPs have a higher cytotoxic effect against HCT-116 colon carcinoma cells at high concentrations, resulting in low viability; viability was increased at 6.25 μL concentration to 100%. At high concentrations, the number of viable HCT-116 cells was decreased after the treatment with the filtered extract of *Fusariumoxysporum* and with TiO_2_NPs. The number of viable cells was increased concomitant to a decrease in the concentration, and the inhibition was decreased concomitant to a decrease in the concentration of the filtered extract of *Fusariumoxysporum* and TiO_2_NPs.

#### 3.5.4. Antitumor Activity of Nanocomposites

**Figure 15:**
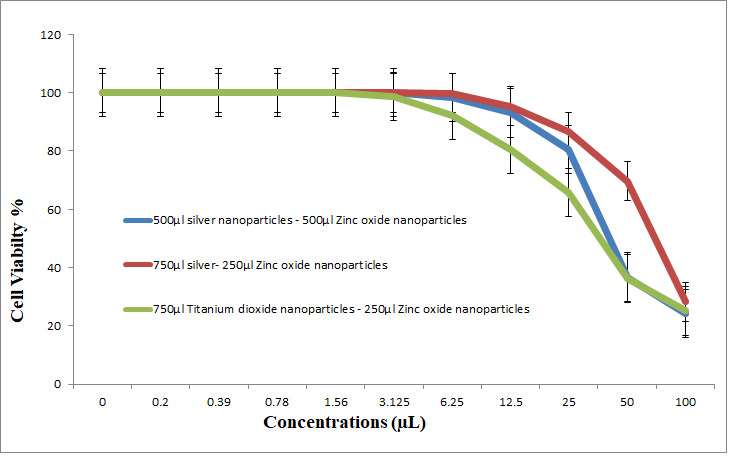
Viability of HCT-116 cells (colon carcinoma cell) (24 h incubation) and treatment with titanium dioxide biosynthesized nanoparticles. Composite 500 μL silver nanoparticles–500 μL zinc oxide nanoparticles; composite 750 μL silver–250 μL zinc oxide nanoparticles; composite 750 μL titanium dioxide nanoparticles–250 μL Zinc oxide nanoparticles.

An antitumor assay for the composites 500 μL AgNPs–500 μL ZnONPs, 750 μL AgNPs–250 μL ZnONPs and 750 μL TiO_2_NPs–250 μL ZnONPs was used to investigate the influence of biosynthesized NPs and capping NCs on cell viability (Figure 15). Cell viability was determined as follows: Cell Viability (%) = (Abs S/Abs C) ×100. The data indicated a dose-dependent decrease at very low concentrations by nanocomposites (500 μL AgNPs–500 μL ZnONPs (IC_50_ = 42.4 μL); 250 μL ZnONPs–750 μL AgNPs (IC_50_ = 73.7 μL); and 250 μL ZnONPs–750 μL TiO_2_NPs (IC_50_ = 38.3 μL)).

In general, nanocomposites are considered to be highly toxic metals (Figure 15). At 3.125 μL, the inhibition was decreased to 0%, 0% and 1.32%, and the viability of the cells became 100%, 100% and 98.68%, respectively. The number of viable cells was increased concomitant to a decrease in the concentrations, and the inhibition was decreased concomitant to a decrease in the concentrations of three types of the nanoparticles.

## 4. Discussion

This study involved biosynthesis of AgNPs, ZnONPs and TiO_2_NP by *Aspergillus* sp. and *Fusarium* [42]. Manipulated conditions favored the activity resulting in the production of metal NPs and NCs. The ability to synthesize AgNPs, ZnONPs and TiO_2_NPs was identified by screening the isolates in the presence of silver nitrate, zinc oxide and titanium dioxide. The presence of AgNPs was observed by using ultraviolet–visible spectra. The surface plasmon resonance band peak in the visible range of approximately 422–428 nm was in agreement with the results of other studies [43]. Ultraviolet–visible spectroscopic studies detected surface plasmon resonance and confirmed the mechanism of the reduction of metal ions and formation of ZnONPs with a peak at 350 nm; thus, our results are in agreement with the data of other studies [44]. TiO_2_NPs had strong absorbance at approximately 360 nm (Figure 1). These results are similar to the data of another study [44]. By comparison, another study [45] reported that TiO_2_NPs synthesized using *Lippiacitriodora* leaf extract showed a strong ultraviolet–visible absorbance peak at 400 nm. Fourteen nanocomposites were tested before and after sonication; the best mixed NCs included 500 μL AgNPs–500 μL ZnONPs, 750 μL AgNPs–250 μL ZnONPs and 750 μL TiO_2_NPs–250 μL ZnONPs, which showed clear peaks on UV [46,47], indicating that AgNPs interacted with DNA; heating demonstrated a hypsochromic shift and widening of absorption spectra [46,47]. In the presence of DNA, a decrease in the intensity was due to partial corrosion of AgNPs [48]. FTIR measurements were performed to identify the presence of various functional groups in the biomolecules. Figure 3A illustrates detection of the peaks corresponding to the bioreduction of silver ions (Ag^+^) and capping responsible for the stabilization of AgNPs [49]. The FTIR spectrum of ZnONPs in Figure 3 showed absorption bands at 592 cm^−1^ and 438 cm^−1^ assigned to the stretching vibration of the Zn-O bonds, which confirmed the formation of ZnONPs [50]. Thus, surface-bound protein molecules act as stabilizers and prevent the aggregation of ZnONPs. FTIR spectra were recorded for TiO_2_NPs (Figure 3A). The band at 1638 cm^−1^ was specified as Amide I, [51] and the band at 1384 cm^−1^ was characteristic for the CH_2_ scissoring vibrations [52]. A peak at 760 cm^−1^ was attributed to the phenyl groups [53]. FTIR spectrum analysis of the composite nanoparticles 500 μL AgNPs–500 μL ZnONPs, 750 μL AgNPs–250 μL ZnONPs and 750 μL TiO_2_NPs–250 μL ZnONPs is shown in Figure 3B–D. The spectra indicated the appearance of the bond peaks corresponding to the O-H stretching at approximately 3925 cm^−1^ [54] and a band peak at approximately 1300 cm^−1^ attributed to Amide III [55]. The Fourier transform infrared spectrum of the Ag-ZnO nanocomposite showed an absorption band peak at approximately 1268 cm^−1,^ corresponding to Amide I of polypeptides [56]. A peak at 1312 cm^−1^ was related to the C-N groups, and this group was related to the appearance of aliphatic amines that act as functional groups [57]. A 2075 cm^−1^ peak was related to the group C≡C carboxylic acids, stretching of alcohols, and ether and ester groups [58]. Fourier transform infrared spectra of the TiO_2_-ZnO nanocomposite with molar ratios different from those of ZnONPs are shown in Figure 3D; the wavenumber range was from 4000 to 400 cm^−1^. The peaks corresponded to the stretching symmetric vibration of the Ti-O-Ti bond, confirmed by the O-Ti-O flexion vibration bond with the band peaks at 550 and 700 cm^−1^ [59]. The peaks at approximately 580 cm and 880 cm^−1^ indicated the Ti-O-Ti symmetric stretching vibration and the Zn-O-Ti group vibration, respectively [60]. The Ti-O and Ti-O-C peaks were at 1518 and 1870 cm^−1^. The interaction between the Ti-O network and organic polymers caused a vibration. The absorption peaks at approximately 400–500 cm^−1^ were assigned to the metal oxygen Zn-O bonds. The peaks at 3500–3600 cm^−1^ indicated the presence of the O-H hydroxyl groups adsorbed on the nanoparticle surface. [49] Transmission electron microscopy of TiO_2_NPs and Ag-TiO_2_ nanocomposites revealed strong interactions between the AgNPs and TiO_2_NPs. The Schottky junction between the metal–metal oxide nanoparticles is formed by the effective interfacial interaction between nanoparticles [61,62]. XRD analysis (Figure 6A) indicated a centered structure of AgNPs based on 2θ at 19.017°, 28.348°, 30.894°, 32.337° and 38.079° corresponding to the (001), (001), (003), (001) and (002) planes, respectively [63]. The patterns show the main peaks corresponding to 2θ at 31.717° and 36.203° in multiple patterns (Figure 6B). The peaks are in close agreement with the data of the literature [64]. The diffraction peaks at 27.389°, 36.043°, 41.205°, 44.007°, 54.425°, 56.734°, 68.960° and 69.966° correspond to the (101), (002), (002), (001), (001), (001), (001) and (002) planes, respectively. Biosynthesized TiO_2_NPs have a more crystalline structure confirmed by the data of Figure 6C. The high-intensity peak 2θ = 27.3° completely matched the crystallographic plane (1 10) of the tetragonal structure anatase phase and is in agreement with the standard database of Philips Analytical and the data of [65]. The formation of silver crystalline clusters on the zinc oxide lattice was indicated by the appearance of silver bands in the XRD diffraction patterns due to the presence of distinct features at 31.788°, 34.442° and 36.271° corresponding to the (001), (001) and (002) reflecting planes, respectively [66]. The X-ray diffraction pattern of ZnO-TiO_2_ nanocomposite arrays was closely related to anatase titanium dioxide [66]. Two new band peaks corresponding to 100 and 101 of the quartzite zinc oxide were detected by Philips Analytical X-ray in agreement with our results (Figure 6).

The data of the antimicrobial activity assays indicated that AgNPs, ZnONPs, TiO_2_NPs and nanocomposites 500 μL AgNPs–500 μL ZnONPs, 750 μL AgNPs–250 μL ZnONPs and 750 μL TiO_2_NPs–250 μL ZnONPs had antimicrobial properties compared with the corresponding controls (filtrate extracts, Figure 7). The antibacterial activity data indicated that AgNPs, ZnONPs and their nanocomposites were good antibacterial agents against Gram-positive and Gram-negative bacteria and showed significant antibacterial ability (9.0 to 29 mm diameter) (Figure 7, Figure 8, Figure 9, Figure 10 and Figure 11). Qing et al. [9] documented an excellent photocatalytic antimicrobial effect of AgNPs compared to that of several nanoparticles. However, the authors in [67] reported that TiOPs had no antibacterial effect. Many authors have reported antibacterial effects of ZnONPs against several isolates, such as *Staphylococcus* sp., [68] methicillin-resistant *Staphylococcus aureus*, [69], *Pseudomonas* sp. [70] and *Escherichia coli* [71]. The mechanism of action of AgNPs is not clearly understood. Most likely, the antibacterial effect of AgNPs is related to mechanisms associated with antibiotics [72]. Generally, antibacterial properties of AgNPs are mainly related to silver cation release from AgNPs, which act as a reservoir for these cations [73]. ZnONPs were recently shown to inhibit the growth of *Staphylococcus epidermis* in a size- and concentration-dependent manner [74]. The results of Figure 7, Figure 8, Figure 9, Figure 10 and Figure 11 indicate a wide spectrum of antimicrobial effects of ZnONPs against several microorganisms. For example, aluminum oxide is toxic towards many cells, although some metal oxides are highly toxic [75]. Moreover, AgNPs toxicity was documented, [76] and titanium dioxide was shown to kill a number of bacteria [77]. Adding metals, such as titanium dioxide, to zinc oxide as a precursor can lead to noticeable results, and the precipitation method used in [28] demonstrated that silver-loaded zinc oxide accounted for an extreme increase in the antibacterial effect of zinc oxide. Silver-loaded zinc oxide is believed to be a new type of precursor for inorganic antibacterial agents [78]. The antibacterial activity of NPs and NCs of Ag-TiO_2_ and Ag-ZnO against Gram-negative (*Escherichia coli* and *Pseudomonas aeruginosa*) and Gram-positive (*Staphylococcus aureus* and MRSA (methicillin-resistant *Staphylococcus aureus*) bacteria is shown in Figure 7, Figure 8, Figure 9, Figure 10 and Figure 11. Ag-ZnONPs are highly efficient based on the comparison of antibacterial activities of Ag-ZnO nanoparticles to those of AgNP-TiO_2_NPs. A significant antibacterial effect against Gram-negative bacteria was shown in the case of both types of doped nanoparticles. Comparison between two types of bacteria, Gram-positive and Gram-negative, indicated that Gram-positive bacteria have a cell wall with a stronger molecular network, which is absent in Gram-negative bacteria; silver ions can penetrate through the cell walls of Gram-positive bacteria [79]. An increase in the silver doping concentration in the titanium dioxide and zinc oxide matrix exponentially reduced the percentage of viable bacteria [80]. Metallic nanoparticles effectively obstruct many microbial species according to in vitro antimicrobial studies [81]. Cancer is a multistep, dangerous and widely distributed disease. Oxidative damage and various chemical, physical, environmental and genetic factors can directly or indirectly induce its development. New and effective agents to control this disease are always needed. Various reports demonstrated that silver nanoparticles have important antiangiogenic properties. Compounds possessing antiangiogenic properties may have antitumor activity because they can block the activity of abnormally expressed signaling proteins [82]. Silver nanoparticles showed higher dose-dependent cytotoxic activity against all cell lines compared to that of onion extract alone [83], in agreement with our results in Figure 12. Many nonspecific effects in subcellular organelles and in the cellular microenvironment may explain the anticancer ability of NPs against HCT-116 cells (colonic carcinoma) with IC_50_ values of 0.7–100 µg/mL. The antitumor effects of unmodified and modified surfaces of ZnONPs and TiO_2_NPs with various diameters are summarized in Figure 13 and Figure 14. The key factors that determine cell viability and genetic alterations in tumor cells include nanoparticle size, aggregation tendency, agglomeration to transform the cell structure and nanoparticle diffusion in tumor microenvironments [84,85]. MNP effects on the cellular microenvironment and tissue physiology have not been well studied. It is necessary to develop effective therapies and obtain an in-depth understanding of anticancer activity of mechanical stimuli [86,87,88,89,90]. Additionally, the increased cytotoxic effect of TiO_2_-ZnO-Au nanocomposites was compared to that of TiO_2_NPs and ZnONPs against Du-145 prostate cancer cells [91]. In addition, MNPs provide improved biocompatibility, better surficial stability, and binding affinity for biomolecule conjugation [91,92,93]. Cantilever bending was detected during recognition and binding of a transcription factor to its target sequence on the cantilever [93,94]. The morphological nanosticks of Ag-Au/ZnO nanoparticles can be accommodated into the cells and can damage cancer cells [95], as illustrated by our result in Figure 15.

## 5. Conclusions

Nanocomposites were synthesized in a fast and facile manner using mechanical alloying. Nanocomposites were observed before and after sonication. Various biosynthesized nanoparticles and nanocomposites with antibacterial properties and antitumor efficiency were produced for applications in several biomedical products, and MNPs and MNPCs were used for electrode modification. Nanocomposites could induce a cytotoxic response and interacted with DNA at concentrations of 500 μL AgNPs–500 μL ZnONPs, 750 μL AgNPs–250 μL ZnONPs and 750 μL TiO_2_NPs–250 μL ZnONPs. Hence, monitoring the toxicity of biosynthesized nanoparticles should be taken into account for safety assessment.

## Figures and Tables

**Table 1:** Mixed ratio nanocomposite (v/v) between Ag, ZnO and TiO_2_ nanoparticles.

No.	Nanoparticles μL		No.	Nanoparticles μL	
1	250 μL AgNPs	750 μL ZnONPs	8	250 μL ZnONPs	750 μL TiO_2_NPs
2	250 μL AgNPs	750 μL TiO_2_NPs	9	333 μL ZnONPs	667 μL AgNPs
3	333 μL AgNPs	667 μL ZnONPs	10	333 μL ZnONPs	667 μL TiO_2_NPs
4	333 μL AgNPs	667 μL TiO_2_NPss	11	250 μL TiO_2_NPs	750 μL AgNPs
5	500 μL AgNPs	500 μL ZnONPs	12	250 μL TiO_2_NPs	750 μL ZnONPs
6	500 μL AgNPs	500 μL TiO_2_NPs	13	333 μL TiO_2_NPs	667 μL AgNPs
7	250 μL ZnONPs	750 μL AgNPs	14	333 μL TiO_2_NPs	667 μL ZnONPs

AgNPs: silver nanoparticles, ZnONPs: Zinc oxide nanoparticles, TiO_2_NPs: Titanium dioxide nanoparticles.

## Data Availability

The data presented in this study are available on request from the corresponding author.
